# Supplementary figures and images for: Proteostasis and lysosomal repair deficits in transdifferentiated neurons of Alzheimer’s disease
Source: bioRxiv. 2025 Jan 13:2023.03.27.534444. Originally published 2023 Mar 27. Preprint. [Version 2] doi: 10.1101/2023.03.27.534444 (PMC10081252; doi:10.1101/2023.03.27.534444)

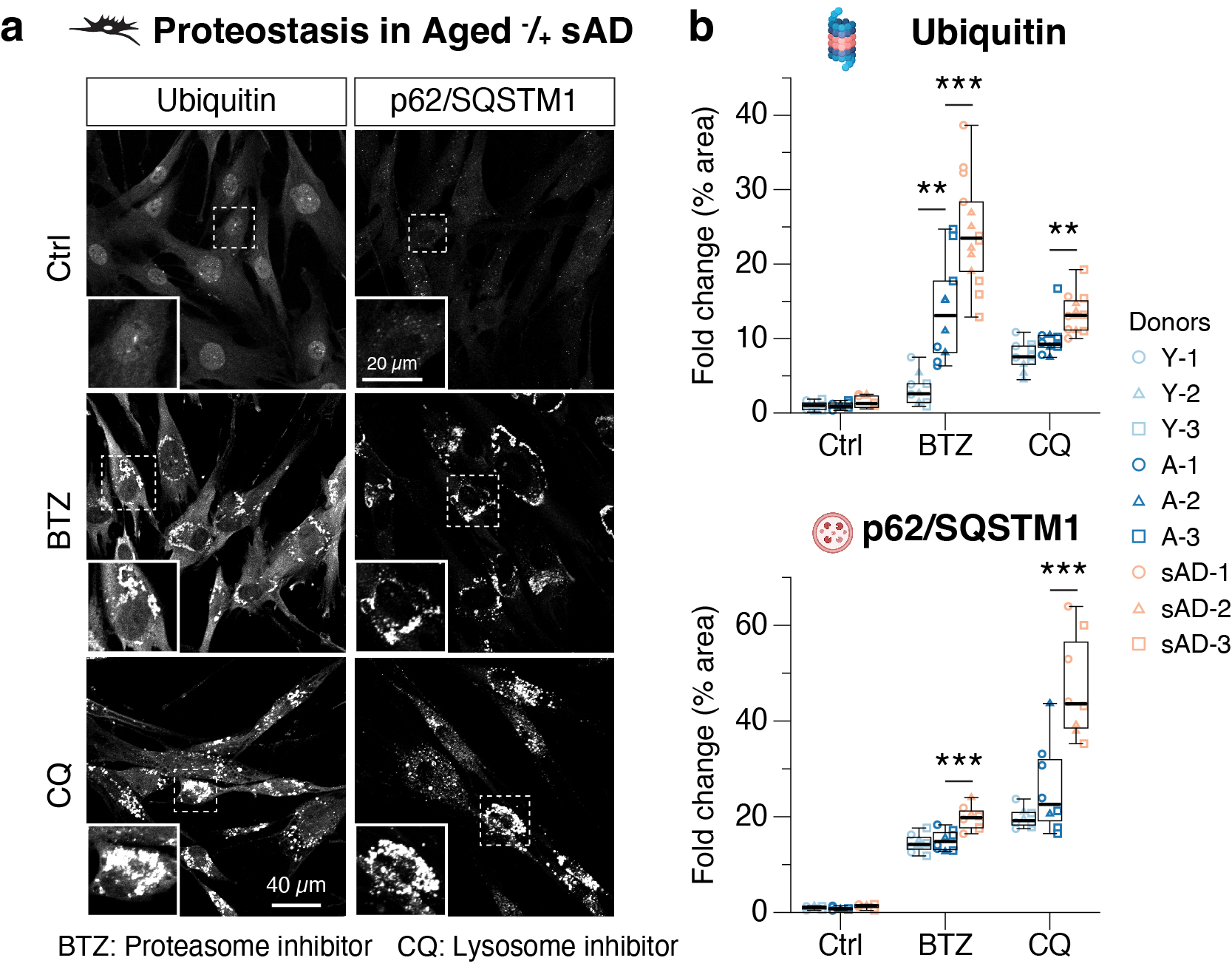

Supplement: Supplement 1 [file media-1.jpg]

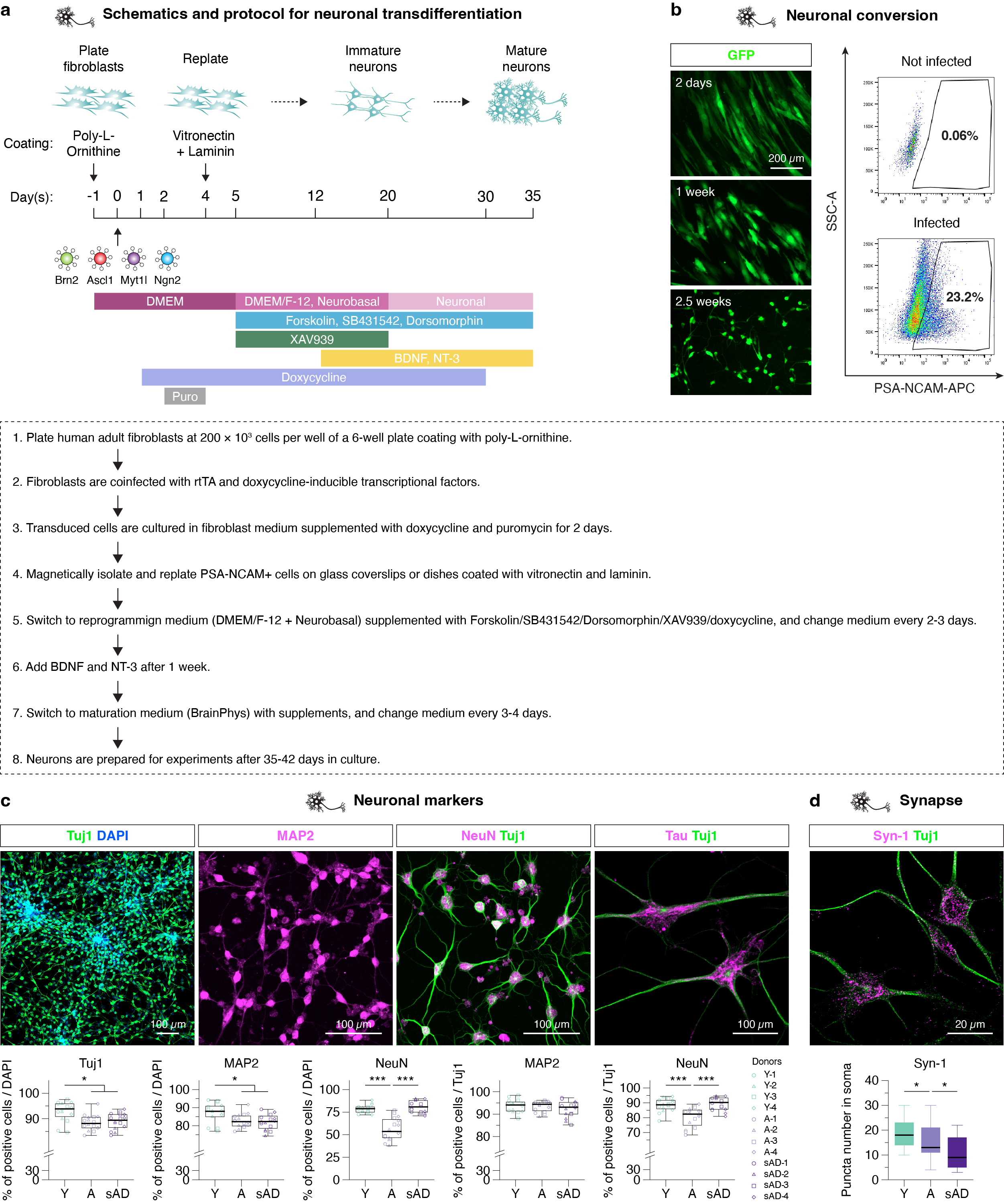

Supplement: Supplement 2 [file media-2.jpg]

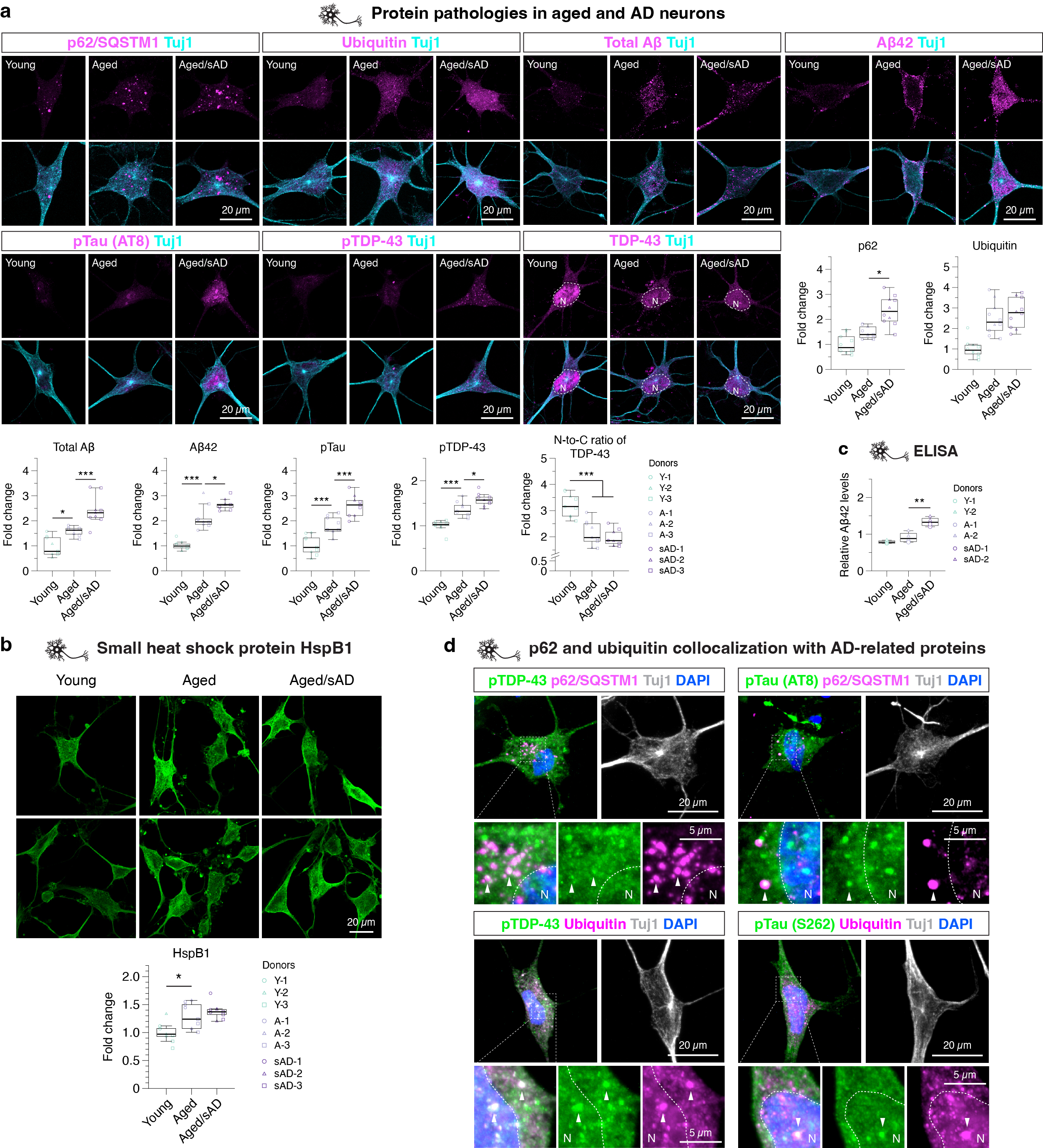

Supplement: Supplement 3 [file media-3.jpg]

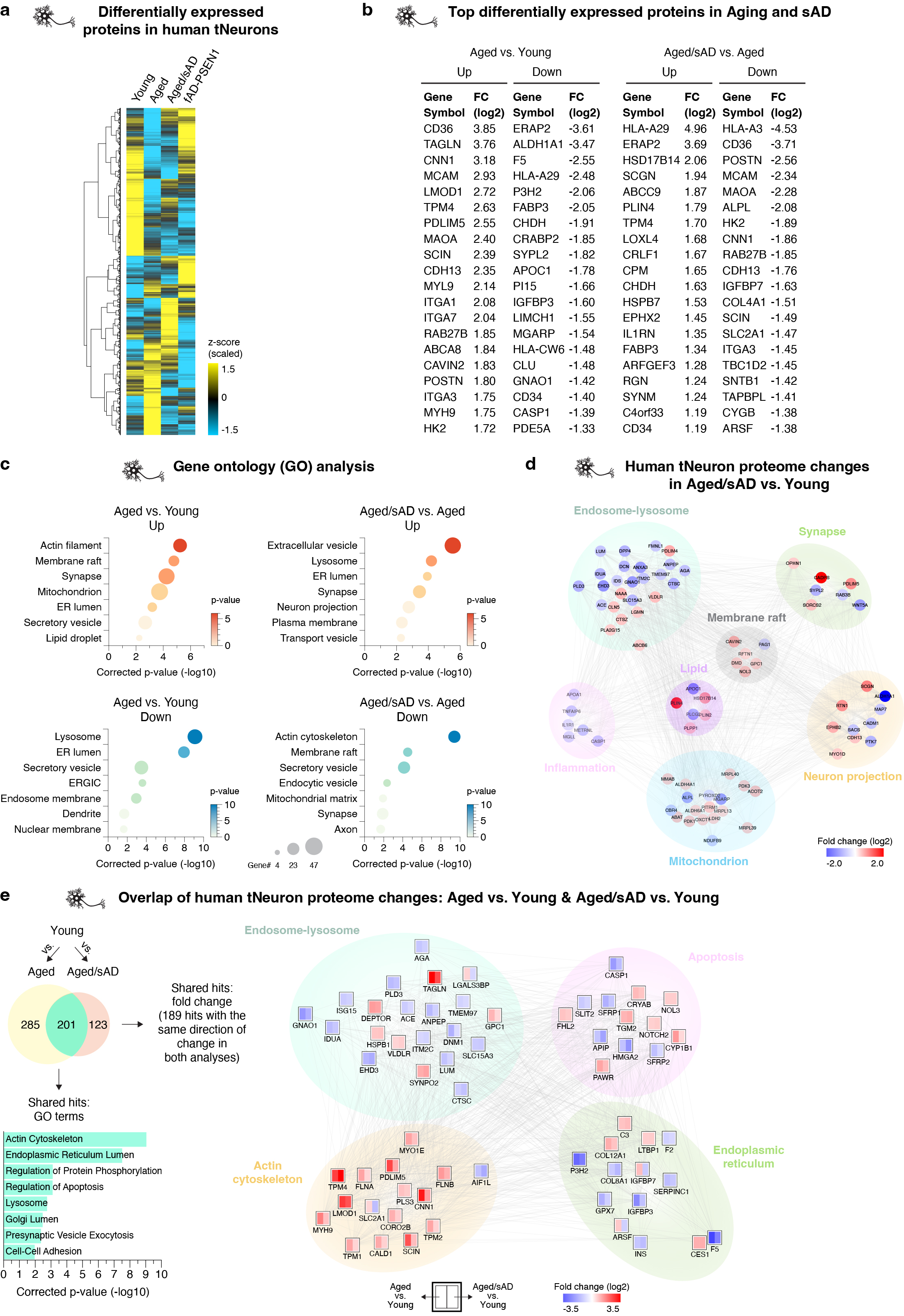

Supplement: Supplement 4 [file media-4.jpg]

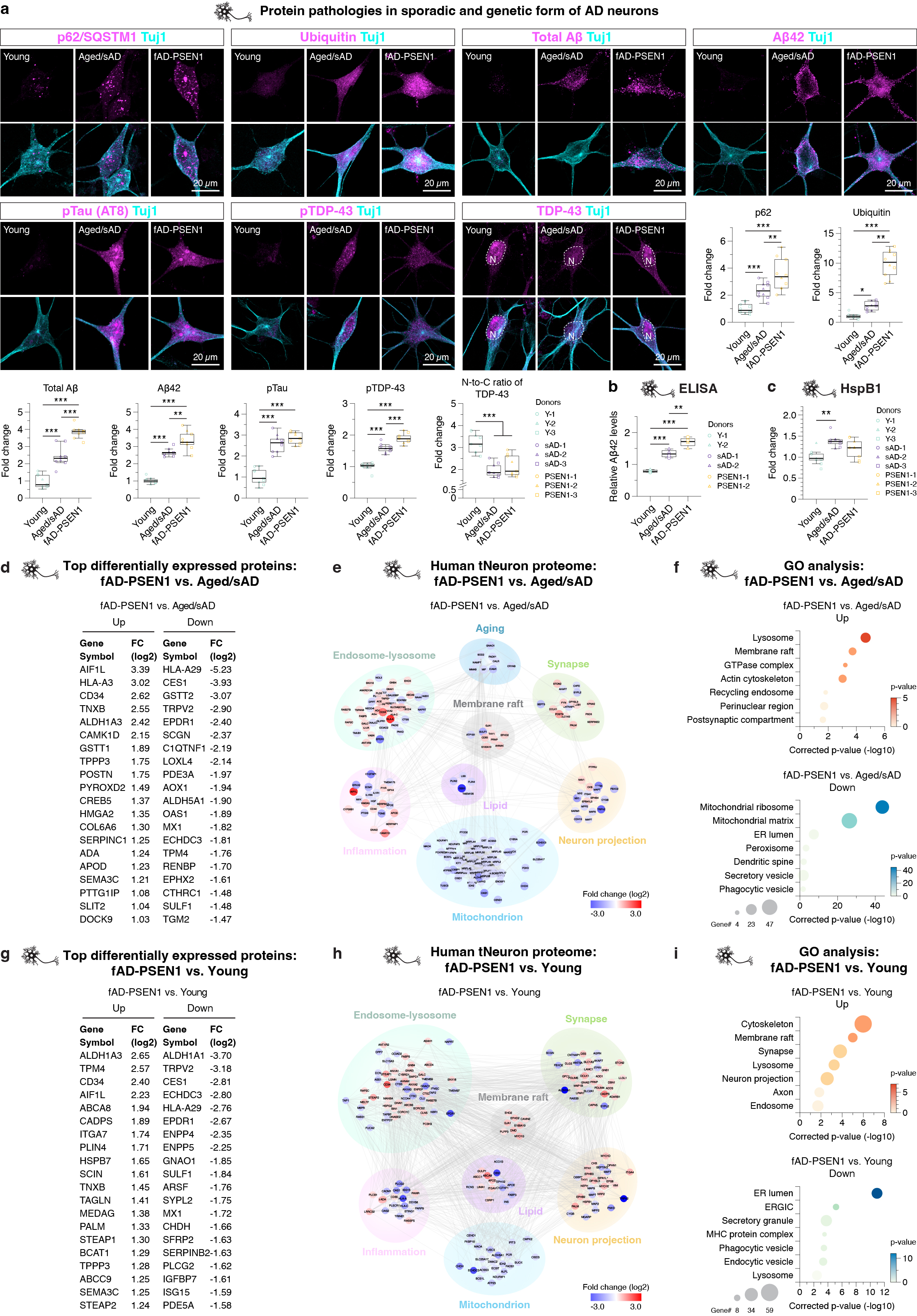

Supplement: Supplement 5 [file media-5.jpg]

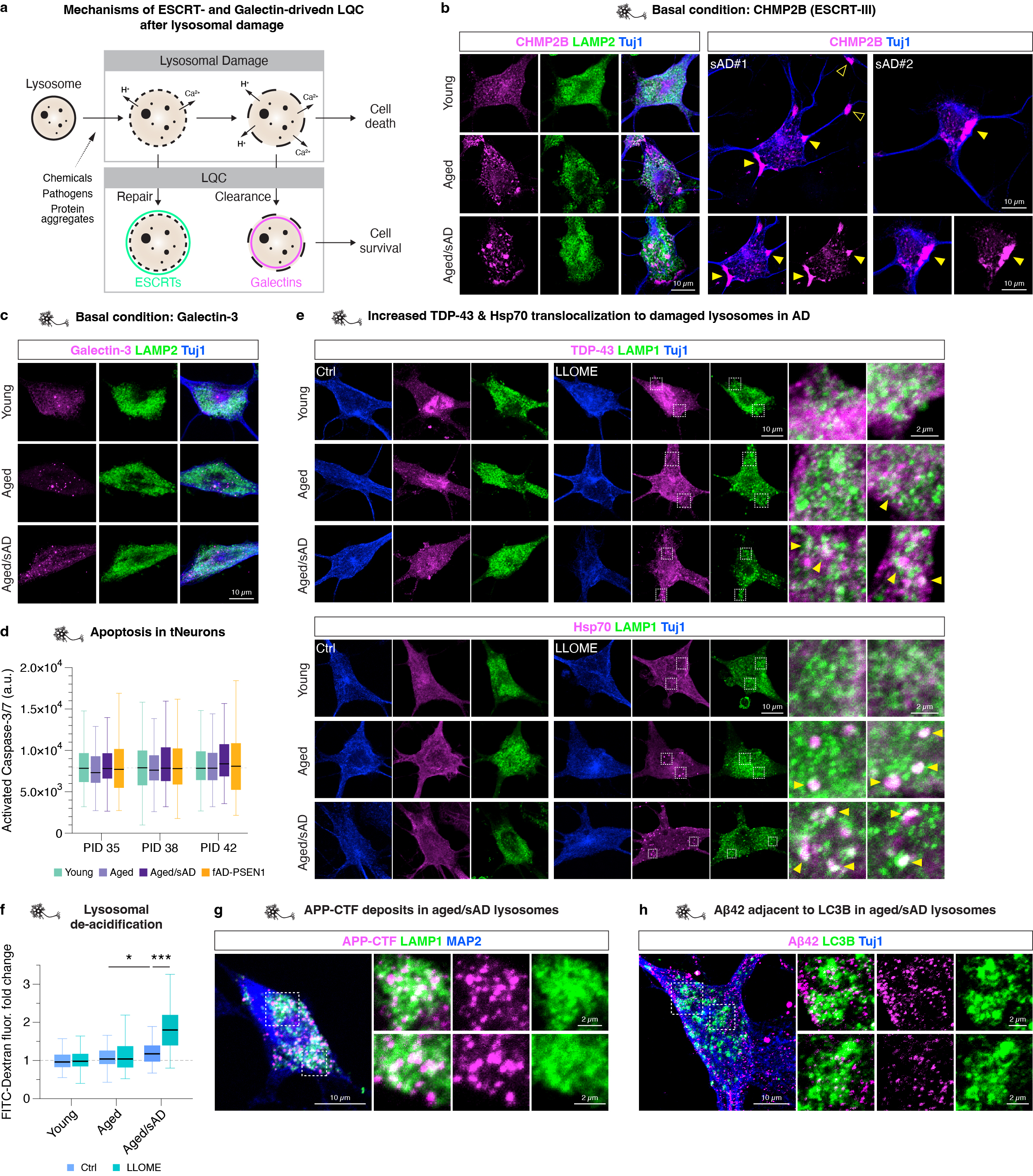

Supplement: Supplement 6 [file media-6.jpg]

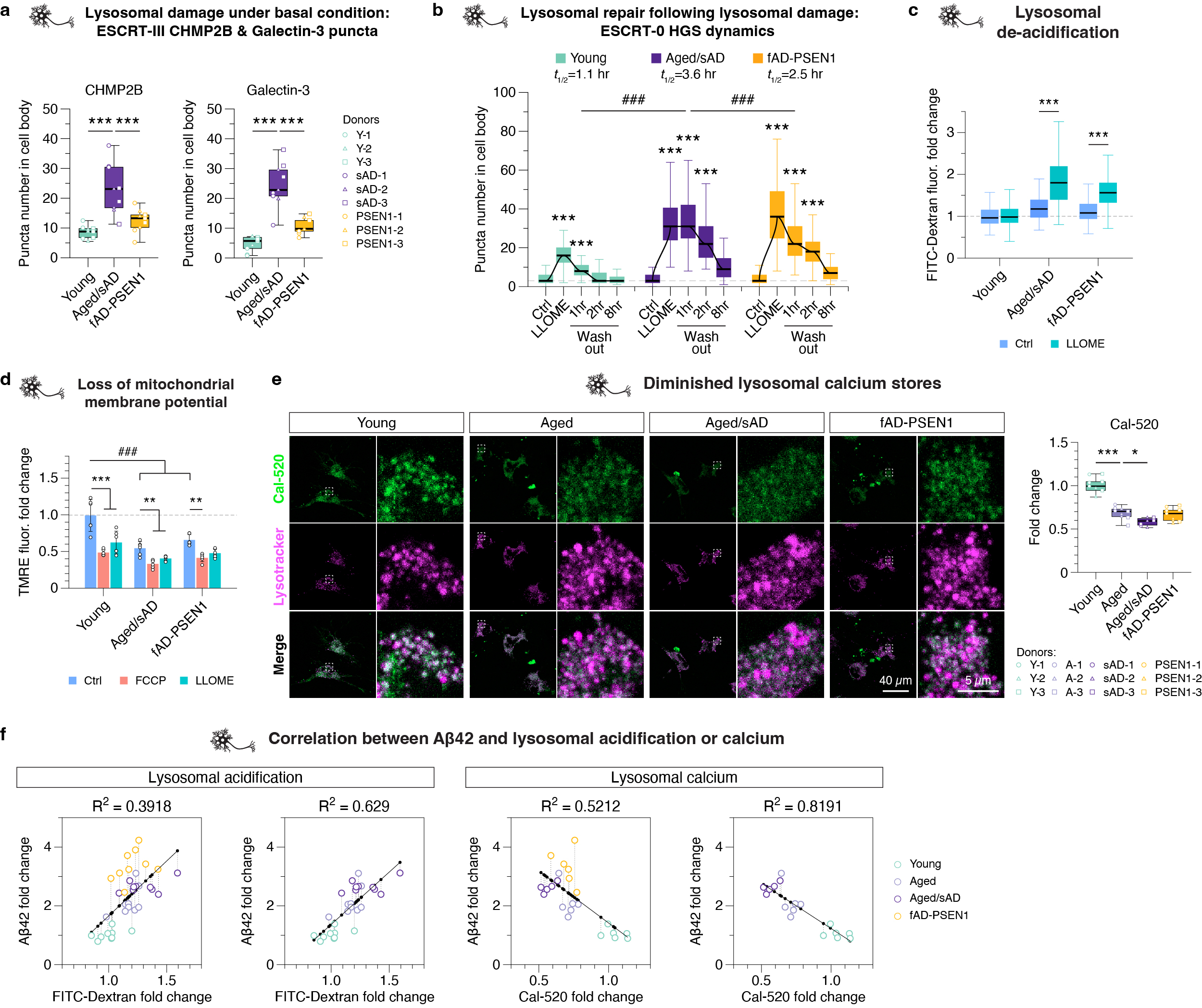

Supplement: Supplement 7 [file media-7.jpg]

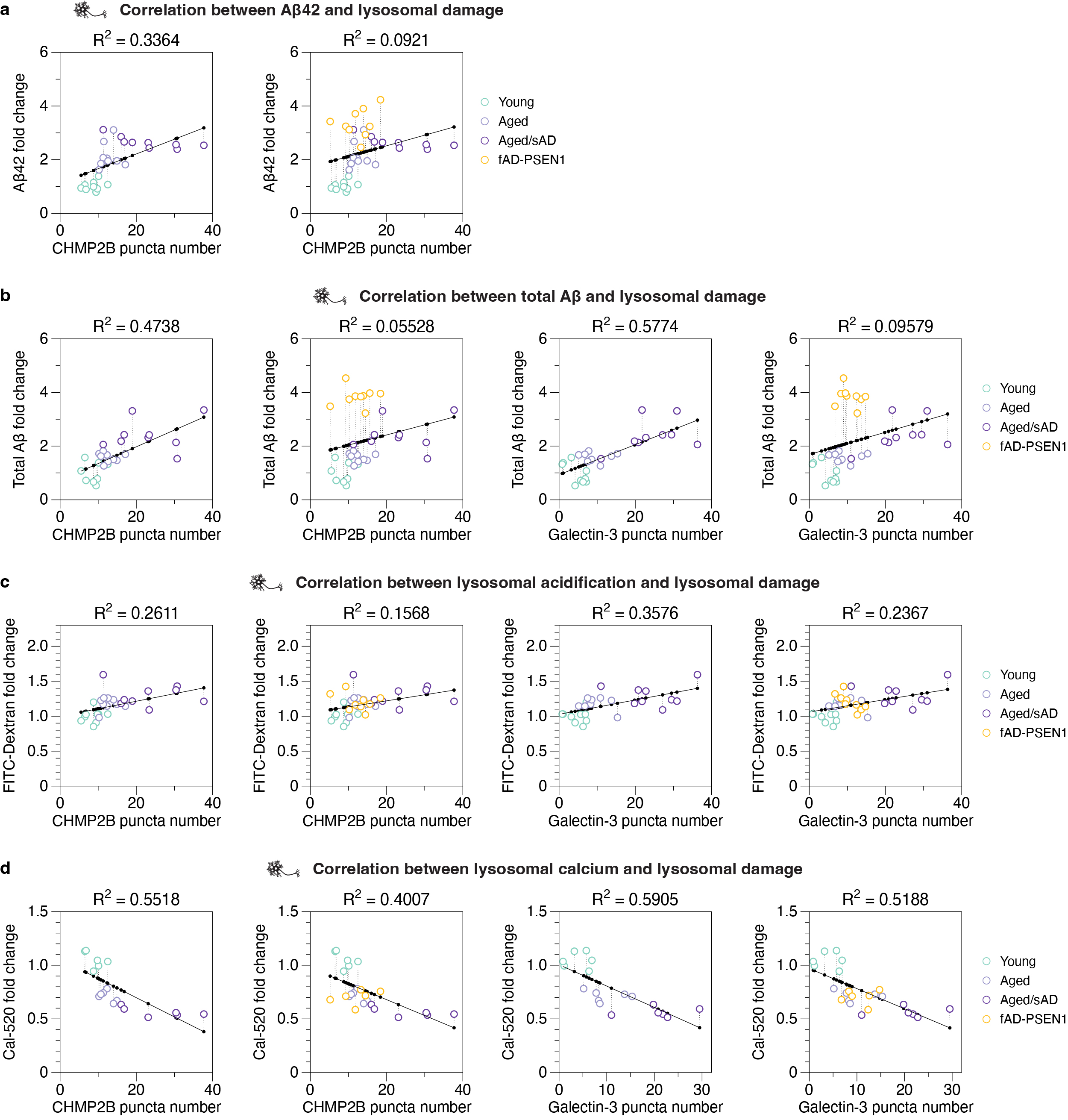

Supplement: Supplement 8 [file media-8.jpg]

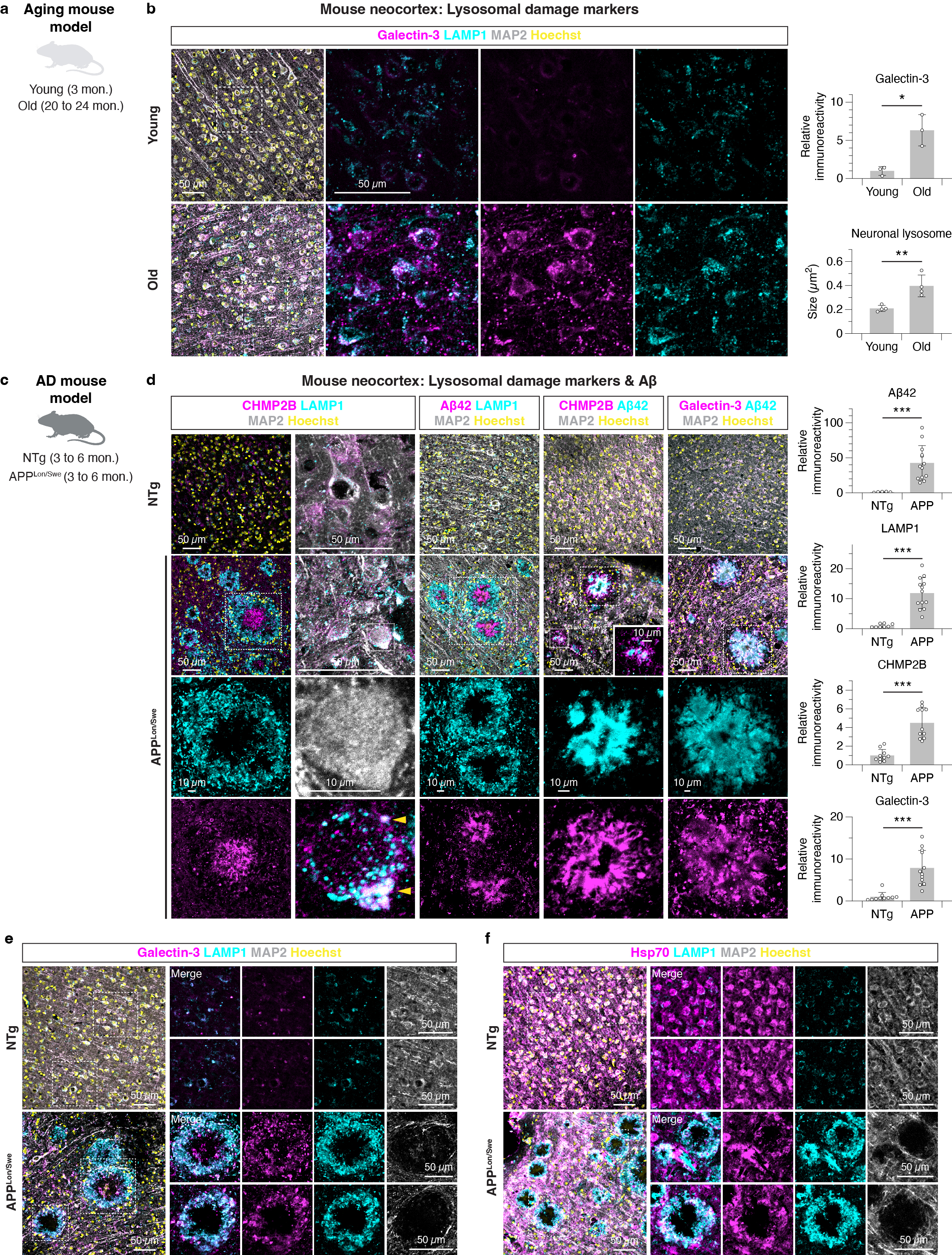

Supplement: Supplement 9 [file media-9.jpg]

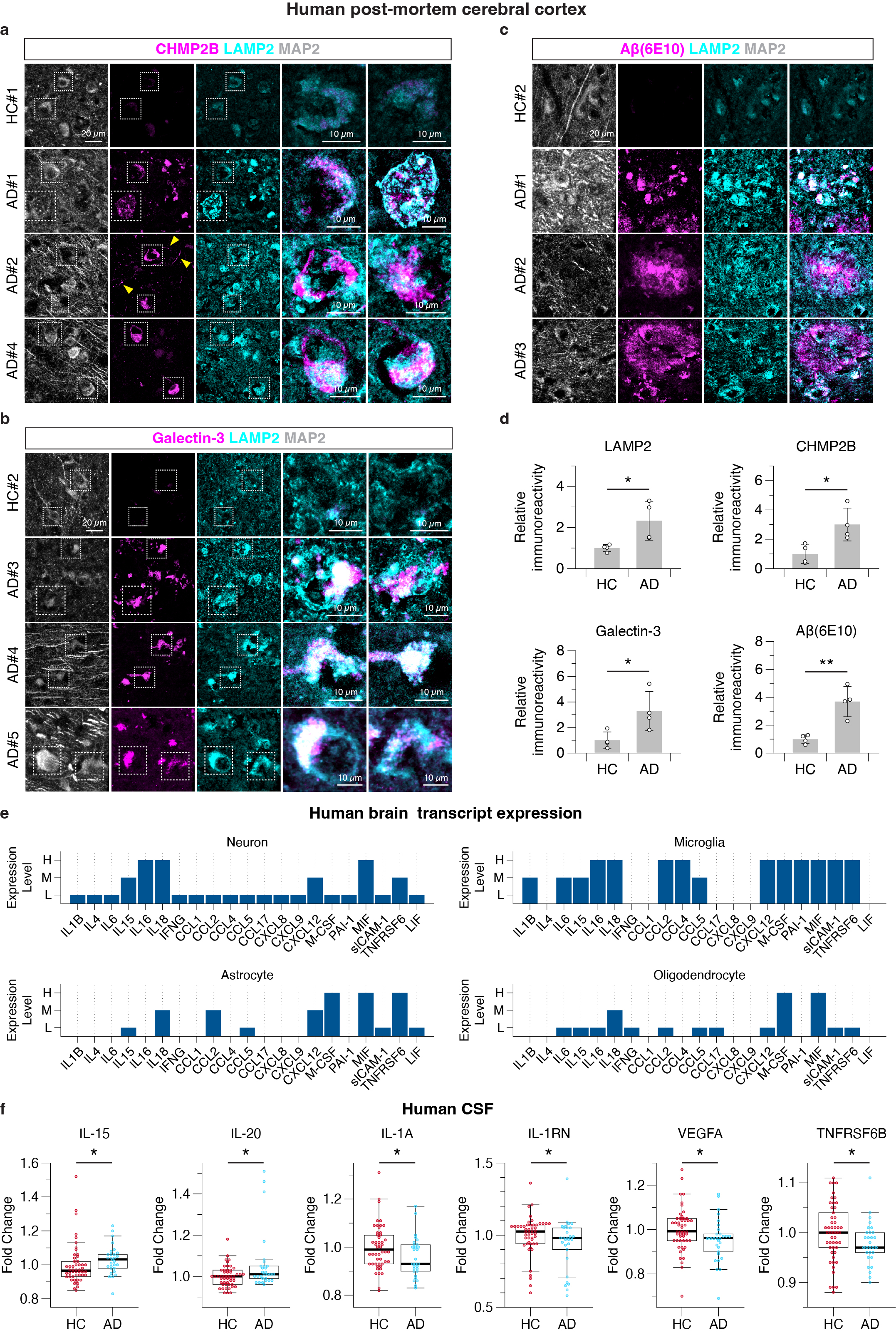

Supplement: Supplement 10 [file media-10.jpg]
