# Supplementary material for: Proteostasis and lysosomal repair deficits in transdifferentiated neurons of Alzheimer’s disease
Source: bioRxiv. 2025 Jan 13:2023.03.27.534444. Originally published 2023 Mar 27. Preprint. [Version 2] doi: 10.1101/2023.03.27.534444 (PMC10081252; doi:10.1101/2023.03.27.534444)
Supplement: Supplement 12 [file media-12.pdf]

Basal condition and chronic lysosomal damage in human tNeurons

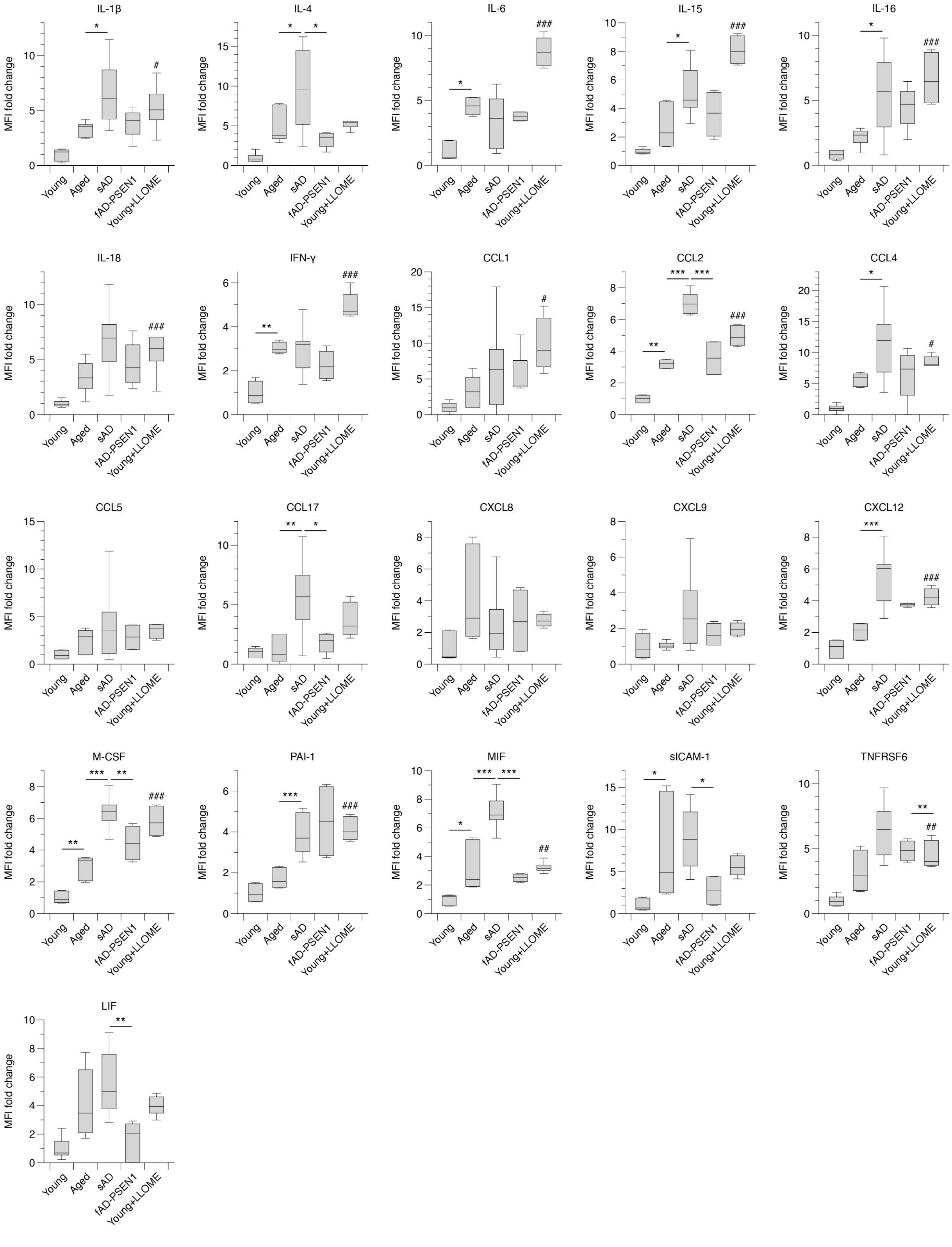

Supplementary Fig.1: Secretion of inflammatory factors from human tNeurons is linked to AD phenotypes.
Plots with statistical analysis of changes in inflammatory factor levels in the conditioned medium from young, aged, aged/sAD and fAD-PSEN1 tNeurons at basal conditions and young tNeurons with the treatment with 0.1 mM LLOME for 7 days, related to Fig. 5d,e.

Data show box-and-whisker plots of inflammatory profiling conducted in technical replicates and 2 to 6 cell lines in tNeurons. Box-and-whisker plots depict 5th, 25th, 50, 75th and 95th percentiles. Differences between groups are compared with One-Way ANOVA followed by Bonferroni post-hoc analysis. *P < 0.05, **P < 0.01 and ***P < 0.001. #P < 0.05, ##P < 0.01 and ###P < 0.001 as compared between young tNeurons with and without LLOME treatment.

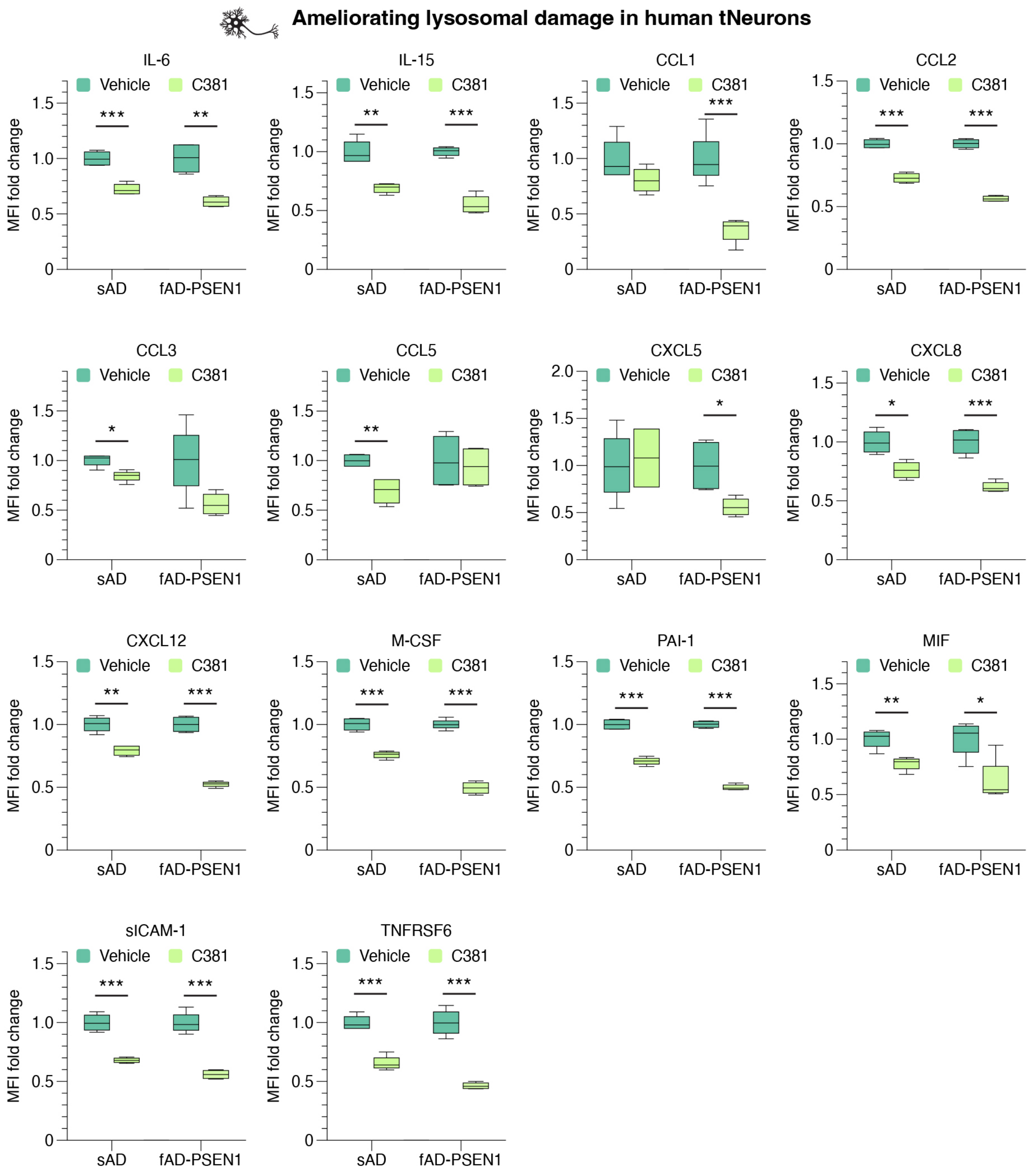

Supplementary Fig.2: Secretion of inflammatory factors from human tNeurons can be reduced by ameliorating lysosomal deficits.
Plots with statistical analysis of changes in inflammatory factor levels in the conditioned medium from aged/sAD and fAD-PSEN1 tNeurons with the treatment of DMSO (vehicle) or 3.1 µM C381 for 7 days as shown in Fig. 5g.

Data show box-and-whisker plots of inflammatory profiling conducted in technical replicates and 2 cell lines in tNeurons. Box-and-whisker plots depict 5th, 25th, 50, 75th and 95th percentiles. Differences between groups are compared with two-sided Student's *t*-test. *$P < 0.05$, **$P < 0.01$ and ***$P < 0.001$.
